# Ultrafast transient absorption spectroelectrochemistry: femtosecond to nanosecond excited-state relaxation dynamics of the individual components of an anthraquinone redox couple†

**DOI:** 10.1039/d1sc04993c

**Published:** 2021-12-17

**Authors:** Sofia Goia, Matthew A. P. Turner, Jack M. Woolley, Michael D. Horbury, Alexandra J. Borrill, Joshua J. Tully, Samuel J. Cobb, Michael Staniforth, Nicholas D. M. Hine, Adam Burriss, Julie V. Macpherson, Ben R. Robinson, Vasilios G. Stavros

**Affiliations:** Department of Chemistry, University of Warwick Coventry CV4 7AL UK v.stavros@warwick.ac.uk j.macpherson@warwick.ac.uk; Molecular Analytical Science CDT, Senate House, University of Warwick Coventry CV4 7AL UK; Department of Physics, University of Warwick Coventry CV4 7AL UK; School of Electronic and Electrical Engineering, University of Leeds LS2 9JT UK; Diamond Science and Technology CDT, University of Warwick Coventry CV4 7AL UK; Department of Chemistry, University of Cambridge Cambridge CB2 1EW UK; Syngenta Warfield Bracknell RG42 6EY UK ben.robinson@syngenta.com

## Abstract

Many photoactivated processes involve a change in oxidation state during the reaction pathway and formation of highly reactive photoactivated species. Isolating these reactive species and studying their early-stage femtosecond to nanosecond (fs–ns) photodynamics can be challenging. Here we introduce a combined ultrafast transient absorption-spectroelectrochemistry (TA-SEC) approach using freestanding boron doped diamond (BDD) mesh electrodes, which also extends the time domain of conventional spectrochemical measurements. The BDD electrodes offer a wide solvent window, low background currents, and a tuneable mesh size which minimises light scattering from the electrode itself. Importantly, reactive intermediates are generated electrochemically, *via* oxidation/reduction of the starting stable species, enabling their dynamic interrogation using ultrafast TA-SEC, through which the early stages of the photoinduced relaxation mechanisms are elucidated. As a model system, we investigate the ultrafast spectroscopy of both anthraquinone-2-sulfonate (AQS) and its less stable counterpart, anthrahydroquinone-2-sulfonate (AH_2_QS). This is achieved by generating AH_2_QS *in situ* from AQS *via* electrochemical means, whilst simultaneously probing the associated early-stage photoinduced dynamical processes. Using this approach we unravel the relaxation mechanisms occurring in the first 2.5 ns, following absorption of ultraviolet radiation; for AQS as an extension to previous studies, and for the first time for AH_2_QS. AQS relaxation occurs *via* formation of triplet states, with some of these states interacting with the buffered solution to form a transient species within approximately 600 ps. In contrast, all AH_2_QS undergoes excited-state single proton transfer with the buffered solution, resulting in formation of ground state AHQS^−^ within approximately 150 ps.

## Abstract 2

A spectroelectrochemical set-up using a boron doped diamond mesh electrode is presented; from ultrafast photodynamics to steady-state, the photochemistry and photophysics of redox active species and their reactive intermediates can be investigated.

## Graphical abstract

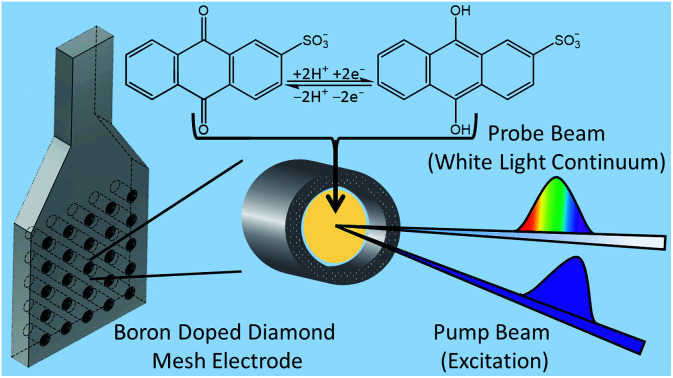

## Introduction

Photoinduced intra- or intermolecular reactions are ubiquitous in many chemical and biological processes,^1,2^ with the latter typically comprising proton transfer or electron transfer (or both, proton-coupled electron transfer)^3^ between the solvent or a second chemical species. Such reactions are of vital importance in many areas including photosynthesis,^4^ photoredox catalysis,^5,6^ photoelectrocatalysis,^7^ electrophotocatalysis,^8^ and photoacidity.^9^ Understanding the transient dynamics of these processes is not only critically important in elucidating mechanistic pathways but also in determining the photostability of different species.

Transient absorption spectroscopy (TAS) is a technique used widely to follow the excited-state dynamics of light-activated complexes. With TAS, measurements at the (sub) ns timescales are possible,^10^ shedding further insight into the critical first step(s) in a reaction mechanism, from excited-state evolution, to either solvent rearrangement,^11^ or intramolecular rearrangement (typically fs to ps),^12^ or proton/electron transfer to solvent (typically ps to ns).^1^ With the use of electrochemical techniques it is possible to switch the oxidation state of a soluble species, simply by control of the electrode potential and direction of electron flow.^13^ Electrochemistry can also be used to access higher reducing and oxidising potentials than possible using chemical oxidants and reductants.^14^ Bringing the two techniques together is an area which is ripe for exploration.^15–18^

There are many processes where the starting species change oxidation state during a reaction pathway, resulting in formation of a less stable, more reactive photoactivated species.^7,19^ In the complementary, burgeoning area of electrophotocatalysis,^8,20^ a reactive radical intermediate is first generated electrochemically and then brought to a higher oxidising or reducing potential by the action of light. Isolating reactive intermediates *via* chemical means for the individual study of early-stage photodynamics is challenging, typically requiring the use of strong chemical oxidising or reducing agents.^14^ Furthermore, the presence of these chemical reagents in the system, unless removed, may complicate dynamic spectroscopic analysis. In contrast, electrochemistry offers an attractive ‘green’ method for the creation of reaction intermediates, *via* controlled oxidation/reduction of the starting species. Moreover, through electrochemical means the product of interest can be generated continuously and there is no need for excess chemical oxidants or reductants. Although it is often useful to consider the impact, if any, of the supporting electrolyte added.^21^ Combined ultrafast TAS (ultraviolet-visible (UV/Vis) or infrared (IR)) – electrochemical measurements thus not only provide routes into the investigation of the photodynamics of isolated reaction intermediates, but also add a new dynamic time dimension to the broad field of spectroelectrochemistry.^16^

To develop this area, a robust experimental set-up is required which can be universally applied. In this work, we introduce an experimental system based on boron doped diamond (BDD) freestanding mesh electrodes combined with TAS, which enable the ultrafast photochemistry and photophysics of the reduced/oxidised states of a redox couple to be investigated. Such electrodes can also find use in more conventional spectroelectrochemical (SEC) measurements. Freestanding BDD has the advantage that it can be laser micromachined^22^ with high accuracy into an appropriate geometry mesh, through which light can pass with minimal scattering. The laser micromachining process is both time efficient and extremely reproducible leading to ease of manufacture. Electrochemically, due to its significantly reduced electrocatalytic activity towards water and dissolved oxygen, it also offers the widest solvent window in aqueous solution, negligible interference from a dissolved oxygen reduction signal, and presents very low background currents.^23,24^ BDD electrodes thus enable discrimination of electrochemical oxidation/reduction processes, *via* voltammetric means, which are often obscured on other electrodes, especially metals. From complementary voltammetric data, the electrode potential for generation of *e.g.* the reaction intermediates, can be established for ultrafast TA-SEC studies.

**Fig. 1:**
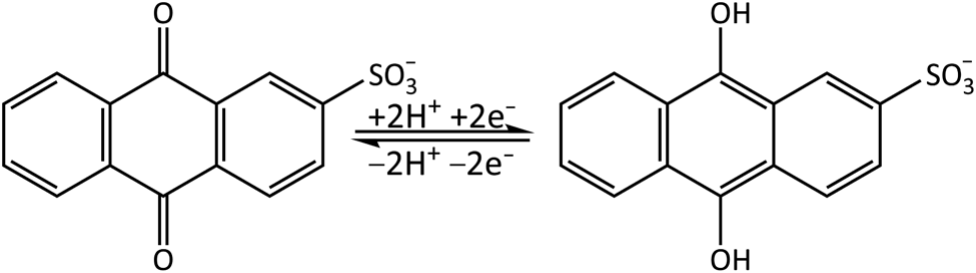
Reversible redox cycle of AQS/AH_2_QS through the acceptance/loss of two electrons (2e^−^) and two protons (2H^+^).

Quinones are important electron acceptors (resulting in the reduced form of the quinone)^25,26^ and play a crucial role in many biological processes.^27^ In this study we investigate the photoactivated dynamics and stability of a model quinone redox couple, anthraquinone-2-sulfonate (AQS)/anthrahydroquinone-2-sulfonate (AH_2_QS). AQS is water-soluble with many applications in the biological as well as chemical fields including as an organo-photocatalyst in the oxidation of alcohols^28^ and nitration of phenol,^29^ a quantifier of reaction lifetimes in surface water bodies,^30^ and in photoelectrochemical cells.^31^ AQS can be electrochemically reduced *via* proton-coupled electron transfer to form its less stable counterpart, AH_2_QS,^32,33^Fig. 1. Using combined ultrafast TA-SEC measurements we showcase how both the oxidised and electrochemically reduced photoactivated states of the quinone can be interrogated at the fs–ns timescale, revealing important information on the photostability dynamics of the AQS/AH_2_QS redox system in aqueous buffer.

## Methods

### Materials and equipment

Solutions were prepared using the following chemicals: sodium anthraquinone-2-sulfonate (≥98% (HPLC), Sigma-Aldrich), sodium acetate trihydrate (laboratory reagent grade, Fisher Scientific), acetic acid glacial (≥99.7% analytical reagent grade, Fisher Scientific), potassium chloride (analytical reagent grade, VWR Chemicals), phosphate buffered saline (PBS) tablet (Fisher Scientific BioReagents™, pH 7.4, 1 tablet/100 mL), sodium carbonate anhydrous (99.5% laboratory reagent grade, Fisher Scientific), sodium bicarbonate (≥99.7% ACS reagent grade, Honeywell Fluka™). Without any further purification, all reagents were used as received. For sample preparation, ultrapure water with a resistivity of 18.2 MΩ cm was used throughout. The solutions were freshly prepared before each measurement, and experiments were performed at room temperature, (20 ± 2 °C), unless otherwise stated.

Steady-state absorption measurements were conducted using a Cary 60 UV/Vis Spectrophotometer (Agilent Technologies, US) and quartz fluorescence cell with a 1 cm path length (Hellma Analytics). Time-resolved measurements were conducted using the TAS instrumentation available at the Warwick Centre for Ultrafast Spectroscopy (https://warwick.ac.uk/fac/sci/wcus/about/). Electrochemical measurements were performed using a PalmSens EmStat3 potentiostat.

### Spectroelectrochemical cell and BDD working mesh electrode fabrication

The 3D printed cell was designed using Autodesk Fusion 360 software (Autodesk, USA) and printed at a 200 μm layer height (Taz 6, Lulzbot, USA) from PET filament (Innofil, USA; green parts) and a FormLabs Form 3 in FormLabs Standard Clear (clear parts). The cell built on a panel holder was designed to fit into the steady-state and the ultrafast instruments (see ESI 1, Fig. S1† for details). A 1 mm path length quartz cuvette (Hellma Analytics) was then cut and adhered to the 3D printed cell using an epoxy resin (Araldite Rapid, USA).

Electrodes were fabricated from freestanding, highly doped (∼3 × 10^20^ boron atoms per cm^3^) electrochemical processing grade polycrystalline BDD (Element Six Ltd, UK),^34^ polished to ∼ nm roughness on the growth face and lapped to ∼μm roughness on the nucleation face with a thickness of 0.25 mm. This material was laser-cut into mesh electrodes with the following dimensions: 6 × 7 mm with holes of 0.25 mm diameter and a 0.35 mm centre to centre space, using a 355 nm Nd:YAG laser micromachining system (E-355-ATHI-O system, Oxford Lasers Ltd. UK) with a nominal pulse-length of 34 ns. Cutting was performed in two passes with a fluence of 350 J cm^−2^. After laser cutting the electrodes were acid cleaned at ∼200 °C in concentrated H_2_SO_4_ saturated with KNO_3_ for 30 min, then rinsed with water and cleaned in concentrated H_2_SO_4_ at ∼200 °C for 30 min, to remove debris introduced during laser micromachining.^35^ After acid cleaning any residual sp^2^ bonded carbon from laser micromachining, was significantly reduced by annealing in air at 600 °C for 5 hours.^35^ A titanium (Ti: 10 nm)/gold (Au: 400 nm) contact was sputtered (Moorfield MiniLab 060 Platform Sputter system) onto the rectangular tail of the electrode and annealed in air (400 °C for 5 h) to create an ohmic contact.^34^ The BDD electrode was connected to a copper wire using a conductive epoxy (Silver Epoxy, Circuitworks, USA), and the connection waterproofed with a layer of epoxy resin (Araldite Rapid, USA).

### Reduction of AQS

Electrochemical measurements were performed using the built cell and a three-electrode system (Pt wire counter electrode, CHI150 Saturated Calomel Electrode (SCE, CH Instruments, Inc) as reference electrode, and BDD mesh working electrode) under an inert atmosphere by bubbling the solution with N_2_. Cyclic voltammetry (CV) measurements were performed on a 1 mM AQS solution (0.1 M acetate buffer pH 4.9, 0.2 M KCl) at a 0.1 V s^−1^ scan rate between −1.0 V and 0.5 V *vs.* SCE (see ESI 1, Fig. S2† for details). A reduction potential of −0.65 V *vs.* SCE was chosen to drive the AQS reduction at a sufficient rate for a time period (30 min) sufficient to convert all the AQS to AH_2_QS. Steady-state spectroelectrochemical experiments were performed by mounting the SEC set-up inside the UV/Vis spectrophotometer. Stability measurements were taken on the electrochemically reduced species (0.2 mM AQS starting material, 0.1 M acetate buffer pH 4.9, 0.2 M KCl) by analysing the UV/Vis spectra over time (see ESI 2, Fig. S3†).

### pH dependence measurements

These measurements were taken using the SEC set-up mounted within the UV/Vis spectrophotometer, using an inert atmosphere and in the following environments: 1 mM AQS in either 0.1 M acetate buffer pH 4.9 (−0.65 V *vs.* SCE) or 0.1 M PBS buffer pH 7.4 (−0.70 V *vs.* SCE), and 0.2 mM AQS in 0.1 M carbonate–bicarbonate buffer pH 8.9 (−0.66 V *vs.* SCE), with all solutions containing 0.2 M KCl. The voltage used for reduction in the different pH solutions was chosen by recording a CV scan at a 0.1 V s^−1^ scan rate between −1.0 V and 0.5 V *vs.* SCE before each experiment. This voltage was applied for 30 minutes before each measurement was taken.

### Transient absorption spectroscopy (TAS)

TAS measurements were performed on 0.1 M acetate buffer pH 4.9, 0.2 M KCl solutions containing either 1.1 mM AQS or electrochemically reduced 1 mM AQS. For AQS, a diaphragm pump (SIMDOS 02) was employed to ensure a fresh flow of sample was delivered constantly to the demountable liquid cell (Harrick Scientific Products Inc.) and a sample thickness of 500 μm was used. For the electrochemically reduced species, the built SEC 1 mm path length static cell was used. Scans of the pH 4.9 buffer solution with no AQS present were taken using the same parameters chosen for the respective molecule of interest, in order to determine potential contributions from the solvent and windows of the Harrick cell or quartz cuvette.

The TAS^36^ set-up has been described in detail in a previous study;^37^ briefly, a Ti-sapphire laser system (Spectra-Physics, Dual Ascend Pumped Spitfire Ace) was used to generate the fs 800 nm (12 W, 1 kHz) pulses, with a nominal pulse width of ≈40 fs. The different pump beams (330 nm at 1 mW and 382 nm at 0.5 mW for AQS and for AH_2_QS, respectively) were produced using an optical parametric amplifier (TOPAS-Prime with UV extension). Pump-probe delays (Δ*t*) between −1 ps to 2.5 ns were used, with changes in optical densities being detected using an Avantes, AvaSpec-ULS1650F spectrometer. The retrieved data was further analysed using the Glotaran software^38^ for the R package TIMP.^39^ A global sequential fit (
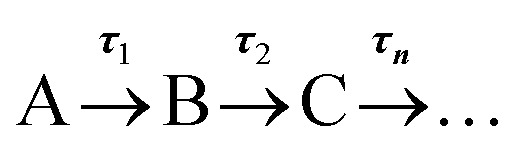
) was employed throughout to extract the lifetimes contained in the TA spectra of our molecules. The standard error for each lifetime was acquired through the Glotaran fit, and the lifetime was reported with twice this standard error. The collected TA spectra were also chirp corrected using the KOALA package^40^ before plotting, for visual clarity. The instrument response duration calculated using the method devised by Kovalenko *et al.*,^41^ retrieved full width half maximum values of ≈130 fs for both the pH 4.9 buffer solution within the Harrick cell (pump at 330 nm) and for the pH 4.9 buffer solution within the 1 mm path length cuvette (pump at 382 nm). Pump power dependence measurements were also performed by varying the output power of the TOPAS. The range of powers used in the power dependence measurements for AQS was 0.15 mW to 1.5 mW and 0.1 mW to 1.0 mW for AH_2_QS, with five-points measurements taken at varying powers. A 10 nm integration window was then taken across a certain spectral window at a given Δ*t* and the log(power) was plotted against the log(signal).

### Theoretical spectroscopy

#### Implicit solvent time-dependent density functional theory (TDDFT)

Initial geometry optimisations were performed using the NWChem Package^42,43^ at the 6−311++G**/PBE0 ^44–46^ level of theory. The continuum solvation COnductor-like Screening MOdel (COSMO)^47,48^ was used in all calculations to implement implicit solvent (water) effects. The first 8 excited states (S_*n*_) were then calculated at the Franck–Condon geometry, using the same level of theory.

#### Triplet state TDDFT

Triplet state transitions were computed using TDDFT in NWChem as above, with the PBE0 hybrid functional and a 6−311++G** basis set. In addition, ΔSCF DFT^49,50^ calculations were also performed using the PBE0 functional, a 6−311++G** basis set, and a multiplicity of 3.

#### Explicit solvent TDDFT

Calculations were performed with ESTEEM.^51–53^ Initial geometry optimisations and DFT and TDDFT ground and excited state calculations^54–58^ on the isolated systems (gas phase, then implicit water solvent) were performed with the NWChem Package. We test a range of functionals, namely LC-ωPBE, PBE, PBE0, LC-PBE0, and CAM-B3LYP, in order to understand the molecular structures and the energy levels involved in their photoexcitation. Molecular Dynamics (MD) simulations^51,52^ using empirical potentials were performed using the GAFF force field in Amber,^59,60^ with FF obtained by using Antechamber for the solute and solvent species. The molecules were embedded in a water box extending 20 Å from the solute molecule, resulting in boxes of approximate size 9400 atoms. MD was performed with a timestep of 2 fs for the heatup (10 000 steps, *NVT* ensemble), density equilibration (50 000 steps, *NPT* ensemble) and equilibration (50 000 steps, *NVT* ensemble) procedures as detailed in the ESTEEM documentation, with *T* = 300 K applied by a Langevin thermostat with a collision frequency of 1 ps, resulting in boxes of sizes around 44 Å cubed. 200 snapshots each spaced by 2000 timesteps were then extracted, separated by 4 ps. Of these, 50 equally-spaced cluster snapshots were extracted from the trajectory. The extracted cluster incorporated the solute and solvent molecules, with any atom of the solvent within 6 Å of any atom of the solute (typically around 400 atoms). The explicit solvent is intended to be sufficient to well-converge the solvatochromic effect of the solvent. Calculations in Fig. S8† compare results for a subset of the results at 3 Å, 6 Å, and 9 Å cluster sizes, and indicate that this has been achieved by 6 Å. The average number of atoms in models of AQS and AH_2_QS in water for *R* = 6 is 385 and 395, respectively; for *R* = 9, for AQS it is 914, and for AH_2_QS it is 917.

From the MD trajectories, the ensemble of explicitly solvated structures is collected and Linear-Response TDDFT calculations are performed on extracted clusters, using the ONETEP (Order-N Electronic Total Energy Package) Linear-Scaling DFT package.^61^ Simulations are performed in a 26.5 Å simulation cell, in which the combined cluster of solute and solvent molecules is surrounded by implicit solvent to minimise any effects from spurious uncancelled dipole moments at the surface of the cluster.^62^ Optimisation of the support functions for the valence and conduction manifolds was performed.^63^ The density and transition kernels were left as dense matrices throughout as truncation was not necessary. Support functions were of radius 5.3 Å and represented with a cutoff energy of 800 eV, spanning to 6 eV above the LUMO. Finally, Linear-Response TDDFT calculations^51^ were performed for the first 12 excitations. The spectra resulting from these calculations was then subjected to a “spectral warp” procedure^64^ as motivated and discussed in ESI 6 to generate the final predicted spectra.

## Results and discussion

### Spectroelectrochemical instrumentation

**Fig. 2:**
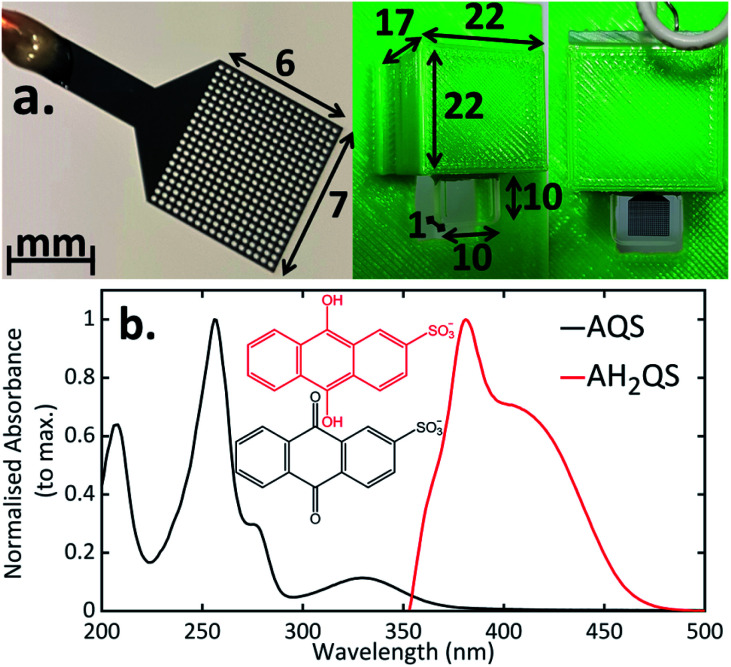
(a) Photograph of the BDD mesh working electrode and the 3D printed cell used in the three-electrode spectroelectrochemical set-up (measurements given in mm); (b) experimental UV/Vis spectra of AQS taken using a 0.2 mM AQS solution in 0.1 M acetate buffer pH 4.9, 0.2 M KCl. For the electrochemically reduced AH_2_QS, a 1 mM initial AQS solution was used (0.1 M acetate buffer pH 4.9, 0.2 M KCl) and a −0.65 V *vs.* SCE reduction potential was applied for 30 minutes before recording spectra, to ensure complete conversion.

Previous TA-SEC studies have employed a variety of electrodes and set-ups. These include: electrodes containing many holes (metal mesh or grid)^18,65,66^ or just a single hole,^15,17^ a flow cell arrangement where the electrode and spectroscopy components are spatially separated,^67^ or a thin layer spectroelectrochemical cell incorporating sufficiently thin planar electrodes.^68^ In the case of mesh/grid electrodes, especially metal electrodes, it is essential that the hole size is made large enough so there is no beam scattering, which can result in the production of charge carriers in the conduction band.^69^ Commercial Pt and Au mesh electrodes are available, however, there are limitations from the sizes of the mesh available, accuracy of the design, and, electrochemically, such electrodes have high background currents and are electrocatalytically efficient towards dissolved oxygen reduction and water electrolysis. This leads to a reduced solvent window in aqueous solution and a detectable dissolved oxygen signal, when scanning cathodically. With a flow cell arrangement,^67^ the range of systems available for study is limited due to the electrochemistry and spectroscopy components of the cell being spatially separated. Whilst sufficiently thin BDD films grown on quartz^70–72^ have been used for thin layer (steady-state) SEC experiments, the manufacturing process can be challenging. The arrangement in Fig. 2a, thus not only enables combined ultrafast TA-SEC measurements, but extends the range of electrodes/geometrical arrangements for conventional steady-state SEC measurements.^16^

### Reduction mechanism of AQS

The CV response for reduction of AQS is shown in ESI 1, Fig. S2,† recorded in a solution containing 1 mM AQS solution in 0.1 M acetate buffer pH 4.9, 0.2 M KCl, and at a scan rate of 0.1 V s^−1^. From the voltammetric response a potential and time were chosen, which were sufficient to drive the reduction of AQS at a rate appropriate for full conversion to reduced products in a reasonable timeframe (−0.65 V *vs.* SCE for 30 minutes). No evidence of AQS was observed in the UV/Vis spectrum (ESI 2, Fig. S3†), indicating complete electrochemical conversion on this timescale. Note, once the reducing potential was removed, or oxygen allowed back into the system, the characteristic absorption signatures of the electrochemically reduced species decreased with time (ESI 2, Fig. S3†).

Fig. 2b shows the UV/Vis data for both AQS (solid black line) and electrochemically reduced AH_2_QS (solid red line). For AQS, in the region of interest, an absorption maximum at 330 nm (ref. 73 and 74) is present. For these studies, we focus on the low energy regions to avoid additional non radiative decay pathways from higher lying excited states when performing the subsequent ultrafast TAS (*vide infra*). The ground state electrochemical reduction mechanisms of quinones have been extensively studied in buffered aqueous media,^33,74–77^ with the many different pH dependent steps and intermediate species described by the ‘scheme of squares’ proposed by Jacq^78^ (see ESI 3, Fig. S4†). In an electrochemical proton-coupled electron transfer reaction, the reduction pathway can be tuned by varying the pH of the environment.^32,33,78^ For this redox couple, when pH < p*K*_a,1_ (= 7.6), *i.e.* in acidic solutions, the doubly protonated AH_2_QS species is formed (2e^−^ + 2H^+^). As the pH is increased, such that pH > p*K*_a,1_ (= 7.6), AHQS^−^ (negative charge denotes loss of a proton) forms, and the mechanism changes to 2e^−^ + 1H^+^. For pH > p*K*_a,2_ (= 11), a 2e^−^ only mechanism dominates, resulting in AQS^2−^ formation.^32,33^ Thus, at pH 4.9, under conditions of exhaustive electrolysis where only the reduced form of the species is present in solution, the spectra observed (solid red line) corresponds to AH_2_QS alone. In particular, an absorption maximum at 382 nm is observed that tails off through the visible region of the electromagnetic spectrum.^32^

### Theoretical analysis of predicted UV/Vis spectrum for AQS

TDDFT calculations using the NWChem Package (6-311++G** basis set; PBE0 functional; implicit solvent) were performed to identify the energy levels involved in the steady-state and ultrafast photoexcitation studies. The transitions observed for AQS are presented in ESI 4, Table S1,† with the peak around 330 nm (Fig. 2b) being assigned to a ππ* transition (S_4_ ← S_0_). The energy of the lowest lying triplet state (T_1_) was calculated using the Delta Self-Consistent Field (ΔSCF) method in the Franck–Condon region, which resulted in an energy of 418 nm. Triplet state vertical TDDFT calculations, although less reliable, indicate that there may be up to five triplet states with energies below the first excited singlet state (S_1_), with both nπ* and ππ* character (ESI 4, Table S1†). Intersystem crossing (ISC) is highly likely, with previous studies also suggesting the formation of a triplet state.^73,79^

### TAS of AQS

**Fig. 3:**
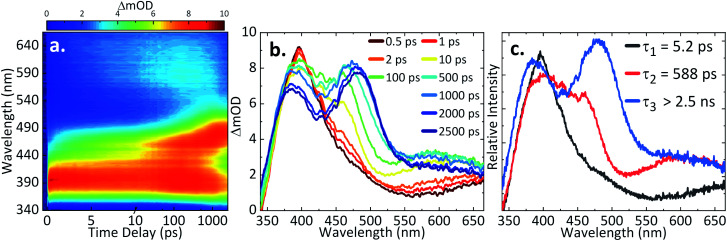
(a) TA spectra of 1.1 mM AQS in 0.1 M acetate buffer pH 4.9, 0.2 M KCl represented as a false colour map showing changes in optical density (ΔmOD) with photoexcitation at 330 nm; (b) different time delays (Δ*t*) chosen for the TA spectra; (c) the corresponding evolution-associated difference spectra (EADS) from the global fit.

In our first step towards unravelling the influence of light on the model redox couple, we studied the dynamical processes following population of the excited state of the oxidised form of the redox couple, AQS. This was achieved through TAS^36,37^ upon photoexcitation at 330 nm. TA spectra were collated at a series of pump-probe time delays (Δ*t*) out to the maximum time-window of our experiments (Δ*t* = 2.5 ns). These are presented as a false colour map shown in Fig. 3a, with individual TA spectra at selected Δ*t* shown in Fig. 3b. The TA spectra initially show an excited state absorption (ESA) feature centred at 395 nm. As this feature decays, two additional features centred at 460 nm and 590 nm are starting to form at Δ*t* > 10 ps. A continuous blue shift of the 395 nm peak as it slowly decays, and a continuous red shift of the 460 nm peak can also be observed. These features are clearly resolved into two separate features, with peaks at 385 nm and 485 nm persisting out to our maximum available Δ*t* of 2.5 ns. Linearity on the photon-induced dynamics was also determined through power dependence measurements (see ESI 5, Fig. S5a†).

To provide more quantitative insight into the dynamical processes involved, we employed a global sequential fit (
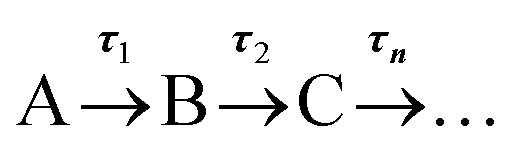
) to extract the lifetimes contained in the TA spectra of AQS. This was achieved through the use of the Glotaran software package.^38,39^ Our sequential fit returned three time-constants with their associated errors, *τ*_1_ = 5.20 ± <0.05 ps, *τ*_2_ = 587.8 ± 9.8 ps, and *τ*_3_ > 2.5 ns, to model the dynamical processes. The evolution-associated difference spectra (EADS)^80^ linked to these time-constants are shown in Fig. 3c, aiding in the visualisation of the transient features within the TA spectra. The associated residuals (difference between raw and fitted data) shown in ESI 5, Fig. S6a,† also serve to indicate the good agreement between the experimental data and corresponding fit.

In order to assign the three time-constants *τ*_1_ (5.20 ps), *τ*_2_ (587.8 ps), and *τ*_3_ (>2.5 ns) to dynamical processes, we draw on earlier photochemical and photophysical studies, and consolidate these with our TDDFT calculations. Previous studies have shown that AQS is promoted to its triplet state shortly after photoexcitation with a ns laser;^73,74,81^ the timescale for the formation of this process was previously unknown due to limitations in the experimental set-up. Close inspection of the absorption spectrum of the triplet state^73,74,79,81^ matches extremely well with the individual TA spectra shown in Fig. 3b for Δ*t* > 10 ps, as well as the EADS for *τ*_2_ (which appears with *τ*_1_ and decays with *τ*_2_), strongly suggesting that the triplet state is formed with a time-constant of *τ*_1_. We add here, the 330 nm excitation peak can most likely be assigned to a ππ* transition involving S_4_ ← S_0_. The rate of ISC may be enhanced by higher lying triplet states (T_*n*_) near resonant with the singlet states or internal conversion from the ππ* states to lower lying nπ* states which can subsequently lead to rapid ISC due to the change in orbital angular momentum.^82,83^ These, along with possible contributions of the heavy-atom (sulfur) in enhancing spin–orbit coupling (SOC)^84^ must contribute to the accelerated ISC we observe. Whether ISC occurs directly from the (likely) initially populated S_4_ → T_*n*_ or whether this is first preceded by internal conversion to the lowest-lying S_*n*_ followed by ISC, in agreement with Kasha's rule,^85^ is not known. From our calculations, we did not identify which of the excited states would exhibit the strongest SOC. Though, in accordance with El-Sayed's rule,^86,87^ an nπ* state is most likely being accessed. Subsequently, AQS remains in its triplet state.

From previous flash photolysis measurements,^74,79^ the photochemistry of AQS was shown to be affected by the environment used, with its excited triplet state forming what is known as transient species B and transient species C (both B and C unidentified); B has a strong, broad feature around 490 nm and C has a weaker feature around 590 nm. Thus, by comparing the time delays > 500 ps (Fig. 3b) with the flash photolysis measurements reported,^74,79,81^ it appears that the triplet state of AQS interacts with the aqueous buffer solution to form transient species B within *τ*_2_ = 587.8 ps, its lifetime persisting beyond the Δ*t* = 2.5 ns time-window of our measurements, with *τ*_3_ > 2.5 ns. In addition, the smaller feature with a maximum peak at 590 nm could be species C, though spectral interferences from other transients may mask its formation. Moreover, given that the environment is slightly acidic, we do not expect to see it forming in high concentration; as previous work suggested, this characteristic feature was more evident in alkaline solutions.^73,74,81^ Importantly, however, it appears that on the timescales of the present measurements (fs–ns) not all of the triplet state reacts with the aqueous buffer environment.

### TAS of AH_2_QS

**Fig. 4:**
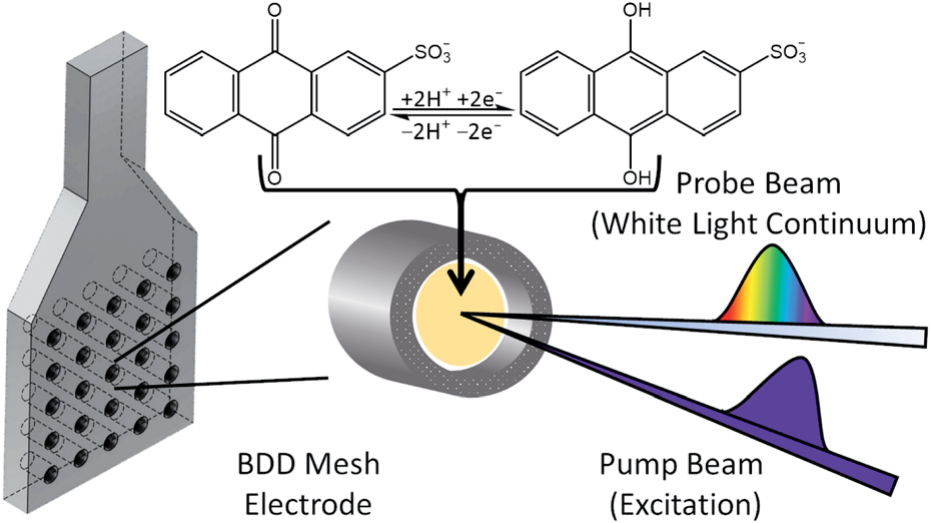
Schematic diagram of the spectroelectrochemical set-up highlighting its use on the TAS instrumentation.

**Fig. 5:**
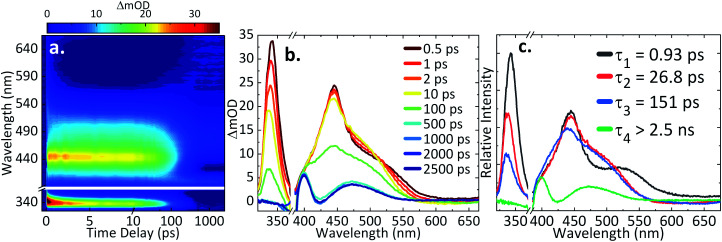
(a) TA spectra of the electrochemically reduced species (AH_2_QS; 1 mM AQS starting material, 0.1 M acetate buffer pH 4.9, 0.2 M KCl, −0.65 V *vs.* SCE), represented as a false colour map showing changes in optical density (ΔmOD) with photoexcitation at 382 nm; (b) different time delays (Δ*t*) chosen for the TA spectra; (c) the corresponding evolution-associated difference spectra (EADS) from the global fit. Note: the AH_2_QS data was plotted by masking the spectral region of the pump leakage (370–390 nm).

For ultrafast TAS interrogation of the dynamics associated with the photoexcited, reduced form of AQS, as for the steady-state UV/Vis experiments, electrochemical reduction was initiated *in situ* by holding the BDD mesh at a potential of *ca.* −0.65 V *vs.* SCE for 30 minutes, to ensure complete conversion of AQS to AH_2_QS, in a buffered pH 4.9 solution. The BDD mesh electrode was coupled to the ultrafast TAS set-up (concept shown schematically in Fig. 4) and the photodynamics of the electrochemically reduced species, AH_2_QS, analysed. The TA spectra collected following photoexcitation of AH_2_QS at 382 nm are presented as a false colour map in Fig. 5a, with individual TA spectra at selected Δ*t* shown in Fig. 5b. Initially (Δ*t* of <1 ps), the TA spectra is comprised of two ESA features. The first feature is centred at 350 nm and the second broad feature, appears to have a main peak centred at 445 nm and a shoulder centred at 525 nm. The feature at 350 nm decays to baseline by Δ*t* = 2.5 ns. In contrast, the features at 445 nm and 525 nm evolve to reveal further features at 400 nm and 475 nm (Δ*t* of >300 ps) which persist beyond Δ*t* = 2.5 ns. Power dependence measurements were also performed to ensure linearity on the photon-induced dynamics (see ESI 5, Fig. S5b†).

The TAS data was once again globally fit using a sequential model (*vide supra* for details) to extract the lifetimes contained in the TA spectra. The EADS linked to these time-constants are also shown in Fig. 5c, aiding in the visualisation of the transient features within the TA spectra. The associated residuals are shown in ESI 5, Fig. S6b.† In comparison to AQS, the data acquired for AH_2_QS is more complex and four time-constants (*τ*_1–4_) were now required to model the dynamical processes involved. A fifth time constant (≈60 fs) accounted for the instrument response (ESI 5, Fig. S7b†) due to contributions from the glass of the cuvette and the solvent itself, but was not taken into consideration when analysing the collected TA spectra. As seen in Fig. 5c, various features are formed in time, with their associated lifetimes and errors of *τ*_1_ = 0.93 ± <0.05 ps, *τ*_2_ = 26.8 ± 1.2 ps, *τ*_3_ = 151.2 ± 2.1 ps, and *τ*_4_ > 2.5 ns.

### pH dependence measurements of the reduction process

**Fig. 6:**
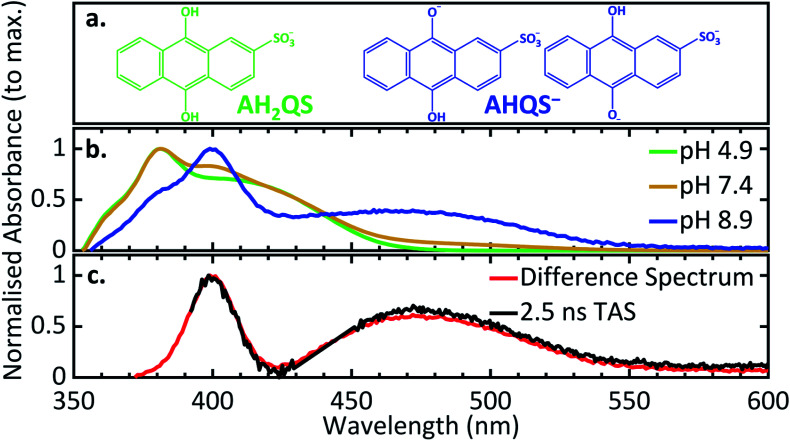
(a) The different protonated forms present at pH 4.9 and pH 8.9; (b) experimental UV/Vis spectra of the electrochemically reduced species at different pH (0.1 M acetate buffer pH 4.9, 0.1 M PBS buffer pH 7.4, 0.1 M carbonate–bicarbonate buffer pH 8.9; 0.2 M KCl added in all solutions; *ca.* −0.65 V *vs.* SCE applied potential for 30 minutes before recording spectra); (c) the UV/Vis difference spectrum between pH 8.9 and pH 4.9 in comparison to the 2.5 ns transient acquired from the TAS data of AH_2_QS at pH 4.9.

In order to deconvolute the complex TA spectra and to understand the observed photochemical response, further steady-state UV/Vis measurements of AQS reduction were performed as a function of solution pH, in the wavelength range 350–600 nm. Given the reported p*K*_a_ values of AQS,^32^Fig. 6a shows the structure of AH_2_QS (reductively formed under acidic conditions, < pH 7.6) and AHQS^−^ (reductively formed under more basic conditions, > pH 7.6). The experiments were also complemented by excited-state calculations in explicit solvent; further information about the techniques used can be found in ESI 6.†Fig. 6b depicts the UV/Vis absorption spectra of the electrochemically reduced species at pH 4.9, pH 7.4, and pH 8.9. A clear difference can be observed between the pH 4.9 and pH 8.9 spectra, with the former associated with AH_2_QS and the latter with AHQS^−^, whilst the pH 7.4 spectrum shows features associated with both. At this juncture, it is worth pointing out the interesting similarity in the pH 8.9 UV/Vis absorption spectrum and the TA spectra for reduced AQS, in pH 4.9 buffer, at selected time delays ≥ 500 ps (Fig. 5b). We return to discuss the significance of this *infra*.

### Theoretical considerations of predicted UV/Vis spectra

The transitions observed from the implicit solvent singlet vertical excitations using the PBE0 functional for AH_2_QS and AHQS^−^, even though less accurate than for AQS, are presented in ESI 4, Table S2;† the lower energy peaks being assigned ππ* (S_1_ ← S_0_) transitions, and the higher energy peaks ππ* (S_2_ ← S_0_) transitions. The triplet state vertical excitation energies in the Franck–Condon region, albeit less reliable, are indicative of a triplet state T_*n*_ with energy below the first excited singlet state (S_1_) being accessed (ESI 4, Table S2†). To lend further weight to the identification of the species contributing to the UV/Vis spectra, *ab initio* explicit solvent theoretical calculations were performed on AQS, AHQS^−^, and AH_2_QS using ESTEEM^51–53^ (see Methods section and ESI 6† for detailed tasks performed). The different nature of the main excitations that contribute to the UV/Vis spectra in AQS and its derivatives, with differing charge-transfer contributions (see ESI 4, Table S1, 2†), mean that there is quite significant variation in peak energies between results for different functionals. We find that LC-ωPBE long-range corrected functional^88^ provided the most accurate results among all the functionals explored (ESI 6, Table S3†), correlating with the expectations that for large conjugated systems containing multiple excitations with different levels of charge transfer character, a range-separated hybrid is necessary for accurate theoretical spectroscopy. However, a fairly substantial blue shift in excitation energies was nevertheless observed,^88,89^ an effect that was also seen in range-separated functionals in previous work performed on a similar molecule, alizarin.^52^

**Fig. 7:**
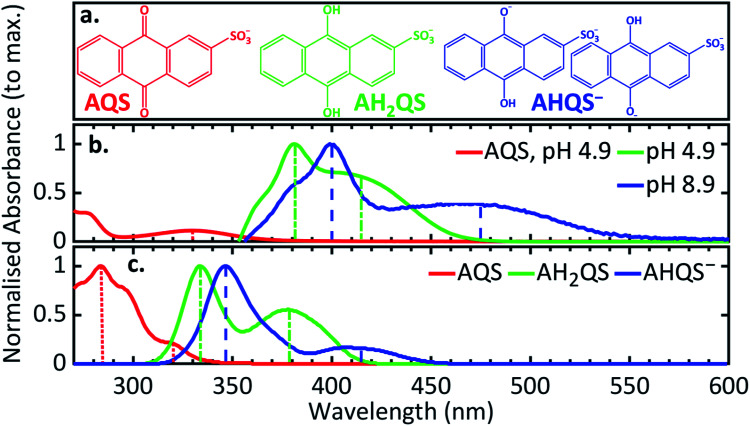
(a) The structures of AQS and the species present at pH 4.9 and pH 8.9; (b) experimental UV/Vis spectra of AQS (0.1 M acetate buffer pH 4.9, 0.2 M KCl) and the electrochemically reduced species at different pH (0.1 M acetate buffer pH 4.9, 0.1 M PBS buffer pH 7.4, 0.1 M carbonate–bicarbonate buffer pH 8.9; 0.2 M KCl added in all solutions; *ca.* −0.65 V *vs.* SCE for 30 minutes before recording spectra); (c) the predicted UV/Vis spectra from the LC-ωPBE explicit solvent TDDFT theoretical spectroscopy with spectral warp corrections applied (see ESI 6†). The vertical lines in (b, c) denote the experimental and theoretical peak positions corresponding to each feature. Note: the two structures studied for the species present at pH 8.9 showed very similar UV/Vis spectra.

The theoretical UV/Vis spectrum from the explicit solvent calculations of AQS was 10 nm blue shifted for the lower-energy peak (320 nm) as seen in Fig. 7a and c, and 28 nm red shifted for the higher-energy peak (284 nm). Interestingly, for AH_2_QS and AHQS^−^, a blue shift was seen for both the higher and the lower-energy peaks; for AH_2_QS the lower-energy peak was shifted by 36 nm and the higher-energy peak by 48 nm (379 nm and 334 nm, respectively), whereas for AHQS^−^, both the lower and the higher-energy peaks (415 and 347 nm, respectively) were shifted *ca.* 60 nm (Fig. 7c). We note here that, due to the close similarity of the theoretical energies in explicit solvent for both structures studied for AHQS^−^, the results were merged into a combined spectrum for this molecule. A consistent blue shift of the theoretically predicted peak positions from the experimental data was observed throughout all the theoretical work in this paper when using range-separated functionals such as LC-ωPBE. This indicates that the different excitation characters contributing to the different peaks, make absolute peak positions more challenging to predict with TDDFT, no matter what functional is utilised. It should be noted, though, that the use of explicit solvent TDDFT has correctly predicted the state order of the transitions for the systems studied, their approximate peak shape and broadening, and the ratios of the heights of the peaks.

### Further discussion of the AH_2_QS TAS data acquired

Taking into consideration the experimental data and the calculations above, we resume our discussion of the TAS data and associated lifetimes of AH_2_QS. At pH 4.9, photoexcitation (at 382 nm) is of the electrochemically reduced species AH_2_QS. Upon population of the excited state (S_*n*_) of AH_2_QS, an evolution out of the FC window with concomitant solvent–molecule rearrangement/relaxation^12^ is described by *τ*_1_ = 0.93 ps in Fig. 5c, with EADS for *τ*_1_ appearing within the IRF and decaying with *τ*_1_. This, in turn, is followed by vibrational cooling from the excited state with a measured lifetime *τ*_2_ = 26.8 ps, with EADS for *τ*_2_ appearing with *τ*_1_ and decaying with *τ*_2_. We add here that the continuous spectral blue-shift in the maximum absorption at 345 nm (Fig. 5b) is also indicative of a vibrational relaxation along the excited state.^90^ These processes appear to take place within the same electronic state.

More interestingly is the origin of *τ*_3_ and *τ*_4_. In Fig. 5c we notice the substantial difference between EADS for *τ*_3_ and EADS for *τ*_4_, which suggests either a new excited electronic state is being populated or the formation of a new species. We draw more insight into this when comparing the individual TA spectra (Fig. 5b), with the spectra in Fig. 6b. As the TA spectra represent difference spectra between the excited and ground states of AH_2_QS, for comparison, a ‘difference UV/Vis spectrum’ has been constructed in Fig. 6c (black line) by subtracting the UV/Vis spectrum at pH 4.9 (AH_2_QS) from pH 8.9 (AHQS^−^), Fig. 6b. The similarity between the 2.5 ns TA spectra (from Fig. 5b) and the “difference UV/Vis spectrum” is striking. The impressive agreement indicates that at this timescale,^1^ photoexcited AH_2_QS has most likely lost a proton and formed ground state AHQS^−^.

Taking into account the system used^73,79^ and the similarity between the TA spectra and the difference UV/Vis spectrum, we can deduce that the most likely cause of proton loss is a solute–solvent (or buffer) intermolecular single proton transfer between the excited state of AH_2_QS and the aqueous buffer solution, generating AHQS^−^; process occurring within *τ*_3_ = 151.2 ps. A caveat is in order here: proton transfer from AH_2_QS could be mediated by a triplet state, as discussed *supra*; we did not attempt to calculate the state arrangement at an ISC geometry in our present calculations but are planning to pursue this in the future to lend further insight. The formed AHQS^−^ persists beyond the time-window of our measurements (Δ*t* = 2.5 ns), its lifetime is assigned as *τ*_4_. These studies highlight the powerful insights that can be gained on early-stage mechanistic pathways from coupling ultrafast TAS with electrochemical methodology.

## Conclusions

A compact spectroelectrochemical methodology based on BDD mesh electrodes, that can be used in a wide range of spectroscopic techniques, from primary (fs–ns) photodynamics all the way through to steady-state measurements, has been successfully developed to assess the time-resolved photochemical and photophysical properties of both the oxidised and reduced forms of a quinone (AQS/AH_2_QS). Whilst in many systems, the reduced form of the electron donating quinone is formed partway through a reaction pathway, we show how electrochemical methods can be used to directly isolate the reduced quinone for photodynamic interrogation. A bespoke freestanding BDD mesh electrode was developed to facilitate these experiments, with the hole size in the electrode, tuned to ensure no light scattering from the electrode itself. Such electrodes also offered low background currents, an extended solvent window in aqueous solution, were easy to fabricate, show reduced electrode fouling, and were also suitable for steady-state UV/Vis spectroelectrochemical measurements.

Ultrafast TAS (UV/Vis) measurements on AQS in pH 4.9 buffer solution highlighted the formation of a triplet state within *τ*_1_ = 5.20 ps, with a proportion of the excited AQS molecules interacting with the aqueous buffer to form different transient species within *τ*_2_ = 587.8 ps. Both pathways had lifetimes persisting beyond Δ*t* = 2.5 ns. Formation of the reduced species, AH_2_QS, was achieved *via* electrochemical reduction of AQS at the BDD mesh electrode. Upon photoexcitation to the lowest energy absorption peak maximum of AH_2_QS (382 nm), excited state relaxation dynamics occurred *via* solute–solvent (or buffer) intermolecular proton transfer within 151.2 ps, between AH_2_QS and the aqueous buffer, which persisted beyond Δ*t* = 2.5 ns. This resulted in the formation of ground state AHQS^−^, at a pH not expected based on p*K*_a_ considerations alone. The TAS data was supported by steady state UV/Vis measurements at different pH values. Explicit solvent TDDFT theoretical calculations were also performed on the proposed structures using the ESTEEM package for the spectroscopic and excited-state characterisation of molecules in a solvent environment. A consistent blue shift of the predicted energies was observed for all molecules, except for the higher-energy peak of AQS which was red shifted. This effect was observed across all functionals studied, LC-ωPBE being the most suitable for this system as the shape, broadening, and height ratios of peaks, along with the state order of transitions were correctly determined.

Whilst the present study has focused on one system, the data showcases the potential for providing routes into a mechanistic understanding of processes involving a change in oxidation state and formation of a photoactivated reaction intermediate. The approach also adds an ultrafast dynamic time dimension to the broad field of spectroelectrochemistry, adding to the suite of techniques available to tackle the many outstanding challenges associated with photoactivated processes.

## Data availability

All the relevant data to this study is presented in the main text and the ESI.† The underlying raw data used in this publication can be accessed *via* the internet through the Zenodo repository at DOI: 10.5281/zenodo.5778925.

## Author contributions

S. G., B. R. R., A. B., and V. G. S. conceived the presented idea. S. G. developed the methodology, built the system, and carried out the experiments. A. J. B., S. J. C., J. T., and J. V. M. contributed in building the spectroelectrochemical cell and the BDD electrode, and in conducting the electrochemical experiments. J. M. W., M. D. H., and M. S. contributed in conducting TEAS measurements. N. D. M. H., M. A. P. T., and S. G. performed the computational calculations. S. G., M. A. P. T., J. M. W., M. S., N. D. M. H., S. J. C., J. V. M., and V. G. S. analysed the results. S. G. and M. A. P. T. designed the figures. S. G. wrote the manuscript with input from all authors. V. G. S., J. V. M., B. R. R., and A. B. supervised the project.

## Conflicts of interest

There are no conflicts to declare.

## Supplementary Material

SC-013-D1SC04993C-s001
