# Supplementary material for: Ultrafast transient absorption spectroelectrochemistry: femtosecond to nanosecond excited-state relaxation dynamics of the individual components of an anthraquinone redox couple
Source: Chem Sci. 2021 Dec 17;13(2):486–96. doi: 10.1039/d1sc04993c (PMC8730129; doi:10.1039/d1sc04993c)
Supplement: SC-013-D1SC04993C-s001 [file SC-013-D1SC04993C-s001.pdf]

Electronic Supplementary Material (ESI) for Chemical Science.
This journal is © The Royal Society of Chemistry 2021

Electronic Supplementary Information for:
Ultrafast Transient Absorption Spectroelectrochemistry: Femtosecond to Nanosecond Excited-State Relaxation Dynamics of the Individual Components of an Anthraquinone Redox Couple

Sofia Goia,[ab] Matthew A.P. Turner,[abc] Jack M. Woolley,[a] Michael D. Horbury,[ad] Alexandra J. Borrill,[a,e] Joshua, J. Tully,[a,e] Samuel J. Cobb,[a,e,f] Michael Staniforth,[a] Nicholas D.M. Hine,[c] Adam Burriss,[g] Julie V. Macpherson,[a]* Ben R. Robinson,[g]* and Vasilios G. Stavros[a]*

a. Department of Chemistry, University of Warwick, Coventry, CV4 7AL, UK
b. Molecular Analytical Science CDT, Senate House, University of Warwick, Coventry, CV4 7AL, UK
c. Department of Physics, University of Warwick, Coventry, CV4 7AL, UK
d. School of Electronic and Electrical Engineering, University of Leeds, LS2 9JT, UK
e. Diamond Science and Technology CDT, University of Warwick, Coventry, CV4 7AL, UK
f. Department of Chemistry, University of Cambridge, Cambridge, CB2 1EW, UK
g. Syngenta, Warfield, Bracknell, RG42 6EY, UK

*E-mail: v.stavros@warwick.ac.uk, ben.robinson@syngenta.com, j.macpherson@warwick.ac.uk

TABLE OF CONTENTS

ESI 1. Spectroelectrochemical set-up 1
ESI 2. UV/Vis Analysis of the electrochemically reduced AQS after air exposure 3
ESI 3. Scheme of squares 4
ESI 4. Time-dependent density functional theory (TDDFT) computational calculations in implicit solvent (water) 5
ESI 5. Power dependence studies, residuals, and solvent measurements for TAS analysis 8
ESI 6. Explicit solvent TDDFT calculations 10
References 13

ESI 1. Spectroelectrochemical set-up

The cell was built for a three-electrode system (reference, counter, and working electrodes) which incorporated the bespoke boron doped diamond (BDD) mesh electrode, Fig. S1.

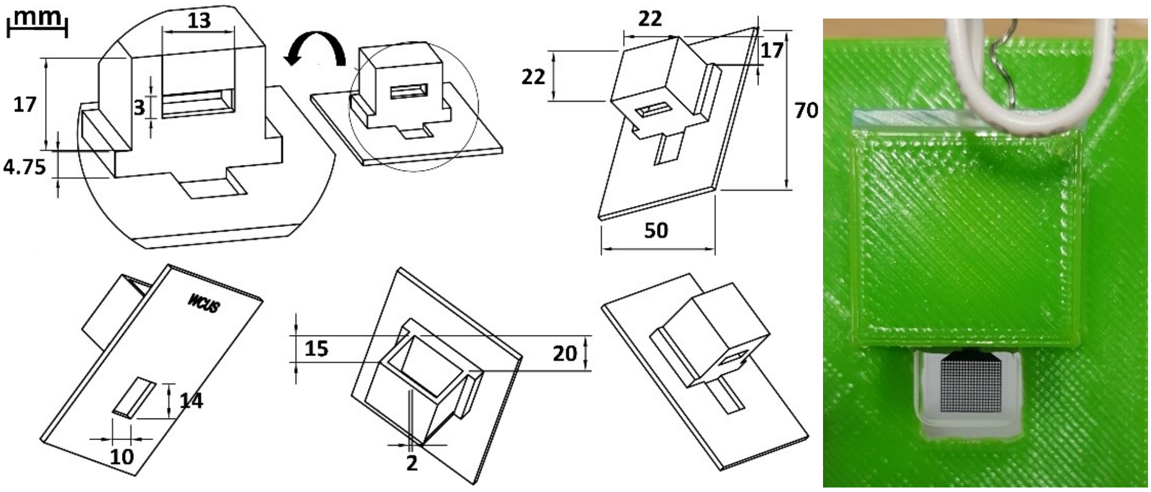

Fig. S1 Diagram of the designed spectroelectrochemical cell using Autodesk Fusion 360 software and a picture of the 3D printed cell containing the BDD electrode (all dimensions given are in mm).

Electrochemical measurements were performed using a three-electrode system comprising a saturated calomel reference electrode (SCE; CH Instruments, Inc), a Pt wire counter (auxiliary) electrode, and the BDD mesh electrode. All measurements were performed under an inert atmosphere by bubbling the solution with N_2. Cyclic voltammetry measurements were performed on a 1 mM AQS solution (0.1 M acetate buffer pH 4.9, 0.2 M KCl) at 0.1 V s^{-1} with the current recorded as the potential was cycled from 0.0 V to −1.0 V and then back to 0.5 V, Fig. S2. The BDD mesh electrode was dipped *ca*. 2 cm in solution and the overall volume used in the cell was 3 mL; the volume in the quartz cuvette was 0.1 mL. The pH of the buffer solution was chosen to promote the two electron two proton reduction of AQS to AH_2QS.[1] A potential was chosen (−0.65 V vs. SCE) which was sufficient to drive the reduction of AQS at a rate appropriate for full conversion of AQS to reduced products in a reasonable timeframe.

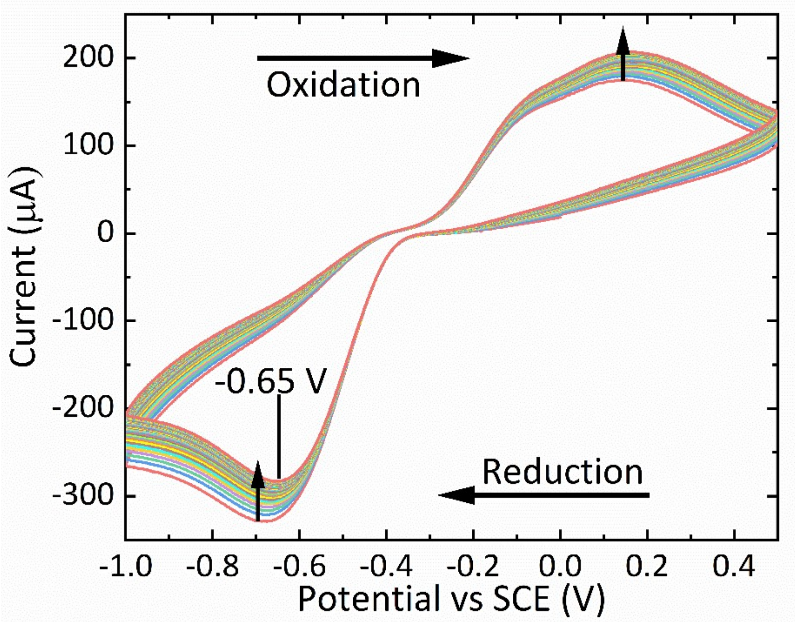

Fig. S2 Cyclic voltammetry measurements (0.1 V s^{-1} scan rate) recorded on 1 mM AQS solution (0.1 M acetate buffer pH 4.9, 0.2 M KCl) using the BDD mesh electrode.

ESI 2. UV/Vis Analysis of the electrochemically reduced AQS after air exposure

Stability measurements were also taken on the electrochemically reduced AH_2QS by analysing the UV/Vis spectra over time under different conditions. The UV/Vis spectrum of the electrochemically reduced AH_2QS at pH 4.9 is shown in Fig. S3 (red line). The UV/Vis spectra were stable at the applied potential and under an inert atmosphere as the reduced species is continually generated. However, after the potential was stopped, the absorbance at 382 nm slowly started decreasing. Moreover, after also ceasing to bubble nitrogen within the cell, exposure to oxygen significantly contributed to the re-oxidation process and AQS was reformed. Two isosbestic points were also formed at 230 nm and at 352 nm as the molecule changes its redox state.

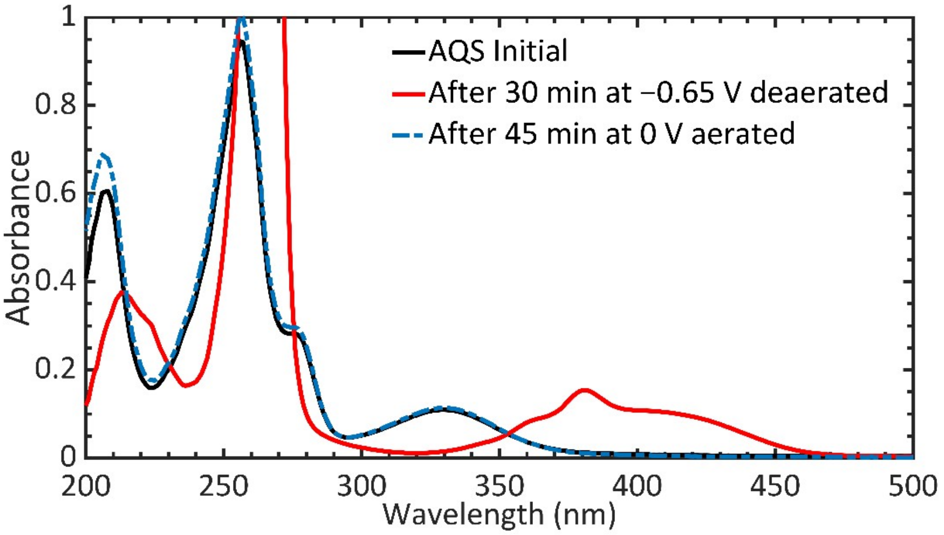

Fig. S3 UV/Vis absorption spectra of AH_2QS upon electrochemical reduction of 0.2 mM AQS solution (0.1 M acetate buffer pH 4.9, 0.2 M KCl) by applying a −0.65 V vs. SCE reduction potential.

ESI 3. Scheme of squares

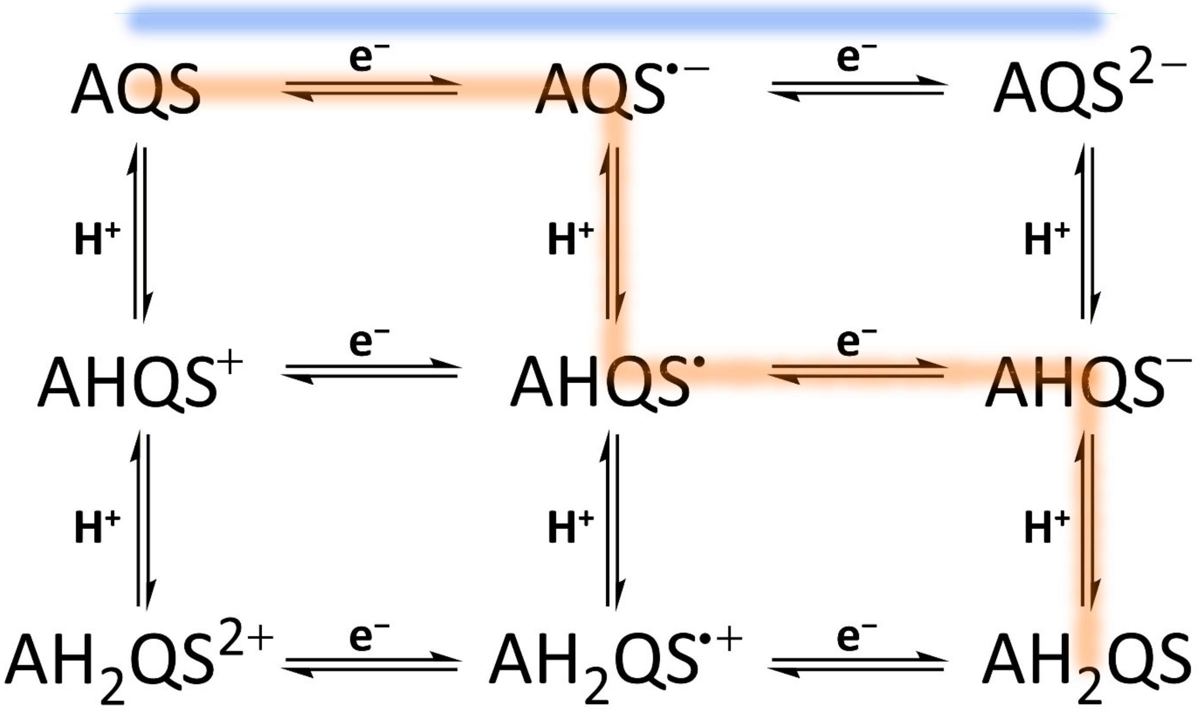

Fig. S4 An overall 'scheme of squares' for the studied AQS/AH_2QS system denoting the mechanistic pathway undertaken at different pH.[1] The orange trace highlights the steps followed in a pH 4–7 environment and the blue trace highlights the steps followed at pH >10, with e^- and H^+ indicating electron and proton transfer, respectively.

ESI 4. Time-dependent density functional theory (TDDFT) computational calculations in implicit solvent (water)

The summary of the singlet vertical excitation energies from the optimised ground state geometry and the triplet vertical excitation energies for AQS are presented in Table S1, along with the orbitals for the first 4 excited states.

Table S1. Calculated singlet and triplet vertical excitation energies and the corresponding orbitals for AQS using TDDFT at the PBE0/6–311++G level of theory**

<table>
<tr><th rowspan="2">Molecule</th><th colspan="4">Singlet</th><th colspan="3">Triplet</th></tr>
<tr><th>State</th><th>Energy (nm)</th><th>Osc. Strength</th><th>Character</th><th>State</th><th>Energy (nm)</th><th>Character</th></tr>
<tr><td rowspan="5">AQS</td><td>S_1</td><td>393</td><td>0.00001</td><td>nπ*</td><td>T_1</td><td>458</td><td>nπ*</td></tr>
<tr><td>S_2</td><td>358</td><td>0.00001</td><td>nπ*</td><td>T_2</td><td>451</td><td>ππ*</td></tr>
<tr><td>S_3</td><td>351</td><td>0.00440</td><td>ππ*</td><td>T_3</td><td>442</td><td>ππ*</td></tr>
<tr><td>S_4</td><td>328</td><td>0.10082</td><td>ππ*</td><td>T_4</td><td>413</td><td>nπ*</td></tr>
<tr><td>S_5</td><td>318</td><td>0.00069</td><td>ππ*</td><td>T_5</td><td>412</td><td>ππ*</td></tr>
<tr><th colspan="8">Orbitals</th></tr>
<tr><td>S_1
n_{HOMO-3}
π^*_{LUMO}</td><td colspan="4">n_{HOMO-3}
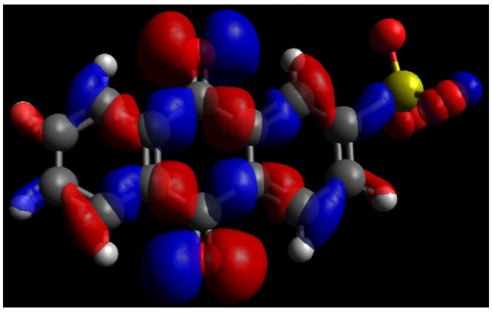</td><td colspan="3">π^*_{LUMO}
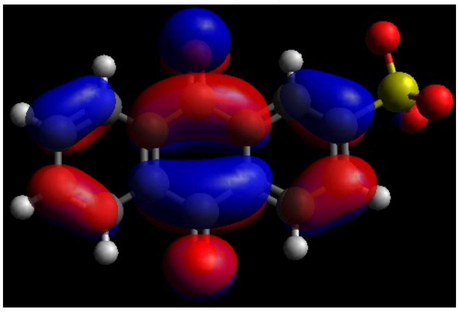</td></tr>
<tr><td>S_2
n_{HOMO-6}
π^*_{LUMO}</td><td colspan="4">n_{HOMO-6}
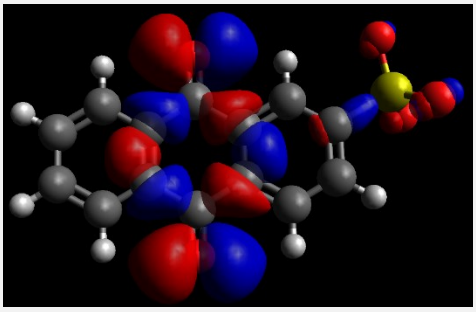</td><td colspan="3">π^*_{LUMO}
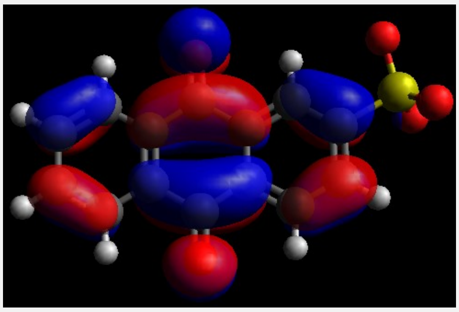</td></tr>
</table>

**S_3**<br>**π_{HOMO}**<br>**π^*_{LUMO}**	π_{HOMO}<br>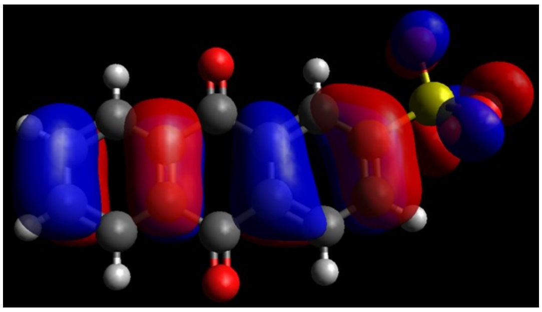	π^*_{LUMO}
**S_4**<br>**π_{HOMO-2}**<br>**π^*_{LUMO}**	π_{HOMO-2}<br>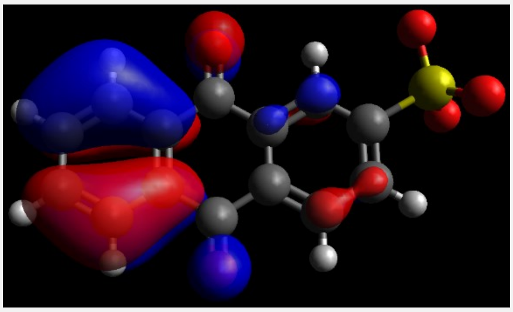	π^*_{LUMO}

The summary of the singlet vertical excitation energies from the optimised ground state geometry and the triplet vertical excitation energies for AH_2QS and $AHQS^-$ are presented in Table S2, along with the orbitals for the first 2 excited states. Triplet state vertical excitation energies indicate that a triplet state with energy below the first excited singlet state (S_1) may be accessed.

Table S2. Calculated singlet and triplet vertical excitation energies and the corresponding orbitals for AH_2QS and $AHQS^-$ using TDDFT at the PBE0/6–311++G level of theory**

State	AH_2QS			$AHQS^-$ (1)			$AHQS^-$ (2)		
	Energy		Character	Energy		Character	Energy		Character
	nm	Osc. Str.		nm	Osc. Str.		nm	Osc. Str.	
S_1	439	0.064	ππ*	512	0.055	ππ*	510	0.059	ππ*
S_2	343	0.131	ππ*	379	0.295	ππ*	374	0.228	ππ*
S_3	297	0.003	ππ*	342	0.003	ππ*	341	0.024	ππ*
T_1	829		ππ*	885		ππ*	902		ππ*
T_2	426		ππ*	518		ππ*	505		ππ*

Orbitals			
	**AH_2QS**	**$AHQS^{-}$ (1)**	**$AHQS^{-}$ (2)**
**S_1 π_{HOMO} π^*_{LUMO}**	π_{HOMO} 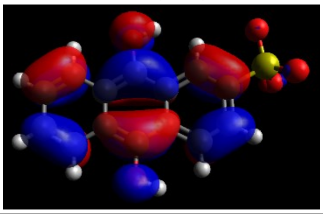	π_{HOMO} 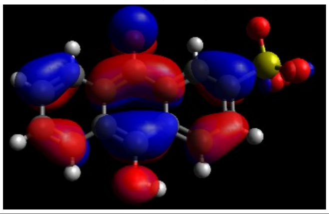	π_{HOMO} 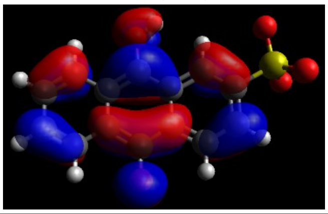
	π^*_{LUMO} 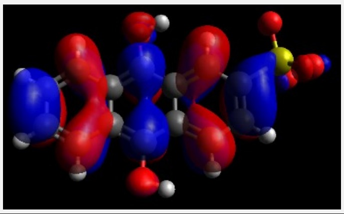	π^*_{LUMO} 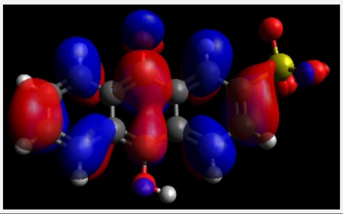	π^*_{LUMO} 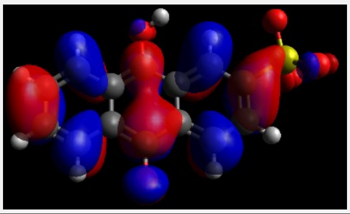
**S_2 π_{HOMO} π^*_{LUMO+1}**	π^*_{LUMO+1} 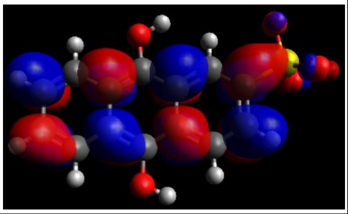	π^*_{LUMO+1} 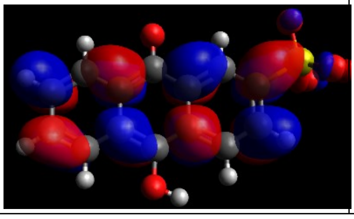	π^*_{LUMO+1} 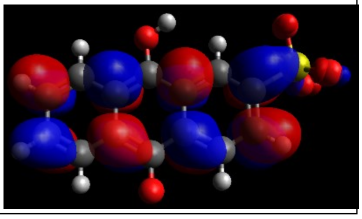

ESI 5. Power dependence studies, residuals, and solvent measurements for TAS analysis

Pump power dependence measurements were performed on AQS and AH_2QS solutions by varying the output power of the TOPAS. A 10 nm integration window was then taken across a certain spectral window at a given Δ*t* and the log(power) was plotted against the log(signal), Fig. S5. These measurements help determine whether a linear dependence on power is present across the spectral window; a slope of ≈1 is indicative of single-photon-induced dynamics, whereas a slope >1 is indicative of multiphoton-induced dynamics. A linear dependence was displayed between the spectral features of the TA spectra and the incident pump power, suggesting single-photon-induced dynamics.

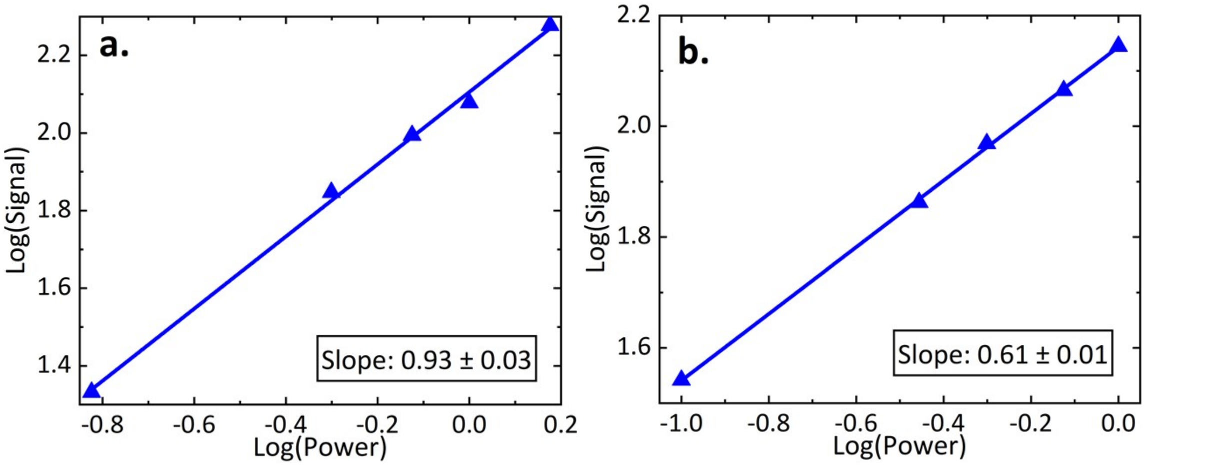

Fig. S5 Power dependence plots for the integrated intensity of **(a)** AQS following excitation at 330 nm using a 10 nm integration window (384–394 nm) and a pump probe delay of Δ*t* = 1 ps; **(b)** AH_2QS following electrochemical reduction and excitation at 382 nm using a 10 nm integration window (342–352 nm) and a pump-probe delay of Δ*t* = 1 ps.

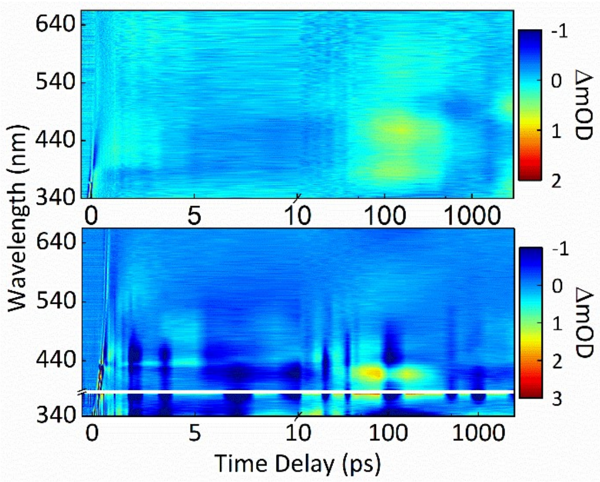

Fig. S6 The residuals between the raw TA spectra and the globally fitted TA spectra for **(a)** AQS and **(b)** AH_2QS. Note: the AH_2QS data was plotted by masking the spectral region of the pump leakage (370–390 nm).

Solvent-only scans of the pH 4.9 buffer solution were taken for AQS and AH_2QS data analysis; at 330 nm pump beam, the Harrick cell with 500 μm spacers were used, and at 382 nm pump beam, the 1 mm path length 3D cell was used. This is done in order to retrieve the full width half maximum (FWHM) of our instrument response function (IRF) which is correlated to the parameters used when fitting the TAS data in Glotaran. The collected TA spectra for the solvent is fitted with a frequency-dependent cross-correlation function (F_{cc}),[2] which also includes contributions from the solvent itself and from the CaF_2 windows of the Harrick cell or the windows of the quartz cuvette. This is calculated using the following equation:

$$F_{cc} \approx exp\left\{ - \left[t_d + t_0(\omega)\right]^2/\tau^2\right\},$$

where t_d is the pump-probe time delay, $t_0(\omega)$ is the temporal overlap between the pump and probe for any given frequency (ω), and τ is the duration of the pump pulse. In Fig. S7a, the 375 nm TA spectrum was fitted and in Fig. S7b the 420 nm TA spectrum, for the pH 4.9 buffer, with their FWHM values determined to be ≈ 130 fs.

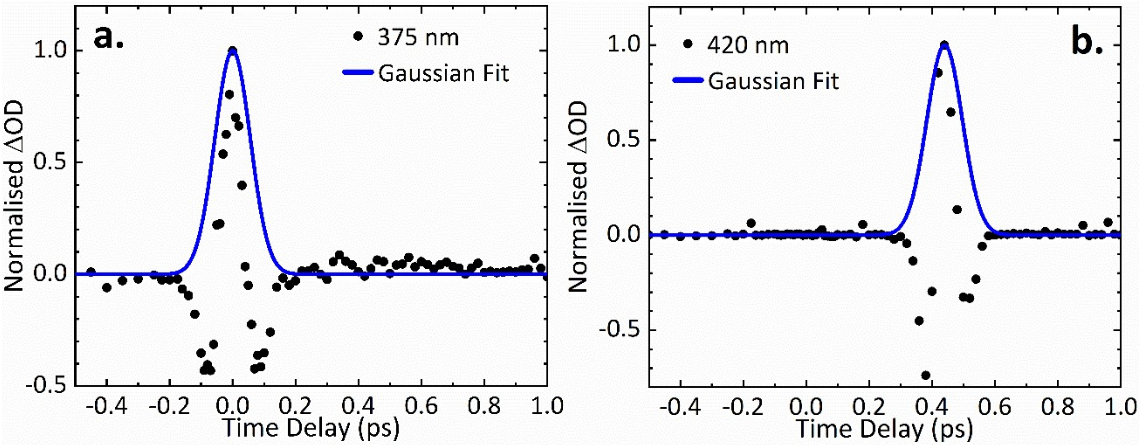

Fig. S7 Solvent-only TAS measurements for instrument response function at 375 nm and 420 nm, respectively, for solvent-only instrument response of: **(a)** pH 4.9 buffer photoexcited at 330 nm (black circles) fitted with the pump-probe cross-correlation function F_{cc} (blue line), FWHM ≈ 130 fs, **(b)** pH 4.9 buffer photoexcited at 382 nm (black circles) fitted with the F_{cc} function (blue line), FWHM ≈ 130 fs.

ESI 6. Explicit solvent TDDFT calculations

In Fig. S8 the results at 3 Å, 6 Å, and 9 Å cluster sizes are plotted to indicate the convergence of the solvatochromic effect of the solvent by 6 Å.

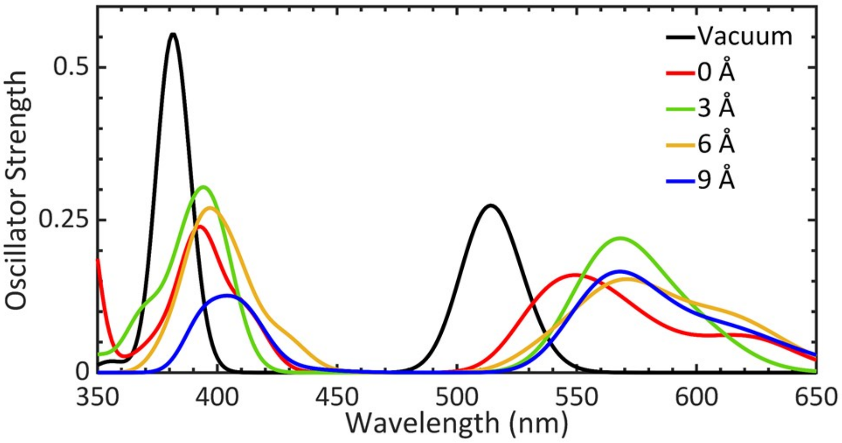

Fig. S8 Comparison between different cluster sizes used to show convergence of the solvatochromic effect of the solvent in explicit solvent calculations in vacuum, at 0 Å, 3 Å, 6 Å, and 9 Å cluster sizes for AH_2QS. Note: the number of snapshots used to plot the spectra were not enough to fully resolve the peak ratios as it is only intended to show peak separations at different cluster sizes.

To make these large-scale calculations feasible, a computationally affordable level of theory is required: we use the GGA-style functional PBE, with a plane-wave-equivalent basis obtained through the support function optimisation process central to ONETEP. The absorption spectra for these extracted clusters are then combined to produce a predicted spectrum for the solvated molecule.

However, three important effects limit the accuracy of this spectrum: (i) the ensemble samples the potential energy surface of the Amber MD trajectories, and therefore inherit any geometry error of the GAFF forcefield description compared to AIMD; (ii) the fact that excitations calculated using the PBE functional can be expected to significantly underestimate the energy of charge transfer states, due to an underestimated HOMO-LUMO gap; and (iii) the fact that TDDFT calculates only unbroadened vertical excitations from each sampled geometry, not incorporating vibronic effects.

In respect to (iii), firstly, it is important to emphasise that the relatively small number of snapshots used per solute (50) is not intended to provide a full sampling of the configuration space or evaluate the peak shape and its broadening, for which we use spectral broadening via a Gaussian of fixed width (0.05 eV) convolved with each excitation.

The peak shift resulting from vibronic effects is not included, but from other work it is possible to estimate that this effect would shift peak positions by no more than 5–10 nm.[3]

Our approach to the combined issues of (i) and (ii) above, is to apply a “spectral warp” as previously used to successfully explain solvatochromism in organic molecular systems.[4–6] The error associated with the functional is dealt with by calculating predicted absorption spectra from equivalent pairs of calculations on each of the 50 geometries of the solute taken from explicit solvent calculations, but with the solvent stripped. One calculation is performed for each geometry using the computationally inexpensive functional suitable for the large-scale calculations (in this case, we used PBE), and one equivalent calculation, on the same geometry, but for the targetted computationally expensive functional (here, options include the LC-ωPBE range-separated hybrid for which the final results are presented in Fig. 7). Warping parameters are calculated through comparison of the spectra, specifically by measuring the energy differences between the two lowest bright excitations, having first verified them to be equivalent excitations in terms of transition origins. This generates the first contribution to the spectral warp parameters.

The error associated with the sampling could only be fully corrected by collecting snapshots from a long-duration MD trajectory performed on a large explicit solvent box using the LC-ωPBE functional, which is computationally unfeasible. Instead, we perform a molecular dynamics calculation on the solute surface in implicit solvent, using a level of theory suitable to capture all relevant geometric effects: in this case the PBE0 functional is well-suited to the task without excessive computational requirements.

To generate a set of snapshots that sample representative geometries on this potential energy surface, *ab initio* QMD calculations were performed with the PBE0 functional, a 6−311++G** basis, and implicit water solvent, for each of the four solute species. Six trajectories of duration > 0.5 ps were generated, using the SVR thermostat to hold the temperature near 300 K. From these, 40 snapshots from each trajectory were extracted, equally spaced from 400 fs of dynamics after a 60 fs equilibration, to produce a total of 240 snapshots of the molecule representing the PBE0 potential energy surface. For 50 of these, vertical excitation TDDFT calculations were calculated for the targeted functionals (range-separated functionals such as LC-ωPBE). The same excited state calculations were repeated for 50 snapshot geometries from the Amber/GAFF trajectory, with the solvent stripped and replaced with implicit water. The second contribution to the spectral warp parameters is the

warp required to take the spectra for the targeted functional from those generated from the geometries sampled by QMD to those generated from geometries sampled by MMD with Amber/GAFF. These warp parameters are mostly quite small, around 0.1–0.2 eV, but significantly improve the comparison of the spectra by, for example, moving the high- and low-energy peaks closer together in energy.

With the energy shifts from these two corrections combined, the explicit solvent spectra undergo shifts based on the resulting warp parameters. The combined spectral warp is applied to the large-scale but cheap-functional spectra to transform them into the spectra that would be expected from large-cluster calculations using the LC-ωPBE functional, on configurations sampled from the PBE0 potential energy surfaces, which would otherwise be prohibitively expensive to perform.[5–7] Results are shown in Fig. 7 for LC-ωPBE, with those for a selection of other functionals shown Table S3 for reference.

Table S3. Calculated explicit solvent singlet vertical excitation energies for AQS, AH_2QS, and $AHQS^-$ at different spectral warp destinations, *i.e.* different functionals

Molecule	Experimental (nm)	LC-ωPBE Energy (nm)	PBE Energy (nm)	PBE0 Energy (nm)	LC-PBE0 Energy (nm)	CAM-B3LYP Energy (nm)
AQS	256	284	405	339	285	308
	330	320	508	392	320	352
AH_2QS	382	334	394	352	330	342
	415	379	528	452	386	420
$AHQS^-$	400	347	410	369	343	354
	475	415	560	483	411	443

References

1 R. S. K. A. Gamage, A. J. McQuillan and B. M. Peake, *J. Chem. Soc. Faraday Trans.*, 1991, **87**, 3653–3660.

2 S. A. Kovalenko, A. L. Dobryakov, J. Ruthmann and N. P. Ernsting, *Phys. Rev. A.*, 1999, **59**, 2369–2384.

3 T. J. Zuehlsdorff and C. M. Isborn, *J Chem Phys*, 2018, **148**, 024110.

4 X. Ge, I. Timrov, S. Binnie, A. Biancardi, A. Calzolari and S. Baroni, *J. Phys. Chem. A*, 2015, **119**, 3816–3822.

5 T. J. Zuehlsdorff, P. D. Haynes, M. C. Payne and N. D. M. Hine, *J. Chem. Phys.*, 2017, **146**, 124504.

6 M. A. P. Turner, M. D. Horbury, V. G. Stavros and N. D. M. Hine, *J. Phys. Chem. A*, 2019, **123**, 873–880.

7 C. Adamo and V. Barone, *J. Chem. Phys.*, 1999, **110**, 6158–6170.
